# Chemotherapy-Induced Nausea and Vomiting: Updates and Recommendations

**Published:** 2018-03-01

**Authors:** Pamela Hallquist Viale

Chemotherapy-induced nausea and vomiting (CINV) has remained a much-feared side effect of cancer therapy. Few pharmaceutical choices were available 35 years ago when I started practice, and when the first serotonin antagonist 5-hydroxytryptamine-3 (5-HT₃) was approved in the late 1980s and brought to market in 1991, oncology professionals celebrated. The combination of steroids and serotonin antagonists made a huge difference for patients undergoing emetogenic chemotherapy, yet CINV remained a significant problem. Thorough patient assessment of pertinent factors helped to improve the control of symptoms as practitioners gained knowledge of particular patient populations at a higher risk for CINV.

The advent of newer targets such as neurokinin-1 (NK1) receptor antagonists and innovative combinations improved the management of this dreaded side effect dramatically. And in yet another direction, we see expanded interest in cannabinoids as another option to improve our control of CINV.

## EVIDENCE-BASED GUIDELINES

Guidelines and recommendations for the management of CINV are available from several organizations.The Oncology Nursing Society (ONS), the Multinational Association of Supportive Care in Cancer (MASCC), and the National Comprehensive Cancer Network (NCCN) are several of the associations that rigorously assess the current data regarding CINV and make clinical recommendations regarding the optimal management of this side effect. And most recently, the American Society of Clinical Oncology (ASCO) updated its own clinical practice guideline regarding the use of antiemetics for CINV (Hesketh et al., 2017).

## ASCO GUIDELINE RECOMMENDATIONS FOR CINV

ASCO used an expert panel to conduct a systematic review of the literature from November 2009 through June 2016. Based on the panel’s assessment of 41 different publications, specific updates for the existing guideline were recommended and included in the guideline for antiemetics in oncology. It is important to note that the guideline included the addition of olanzapine (an atypical antipsychotic proven to be useful for patients receiving highly emetogenic chemotherapy or for those patients experiencing breakthrough nausea and vomiting despite initial antiemetic therapy; Hesketh et al., 2017). The definition of highly emetogenic chemotherapy includes adult patients treated with cisplatin or other highly emetogenic single agents and adult patients treated with an anthracycline and cyclophosphamide combination.

Changes in the control of moderately emetogenic chemotherapy include a recommendation for three-drug combinations (an NK1 receptor antagonist, a 5-HT₃ receptor antagonist, and dexamethasone) for patients treated with carboplatin with an area under the concentration-time curve ≥ 4 mg/mL per minute. Patients receiving moderately emetogenic chemotherapy other than the carboplatin dose above should receive a two-drug combination of a 5-HT₃ receptor antagonist and dexamethasone, and patients treated with agents known to cause delayed nausea should be offered dexamethasone on days 2 and 3 following initial therapy.

Additional recommendations for the control of low emetic risk agents include the use of a 5-HT₃ receptor antagonist or a single dose of dexamethasone (8 mg) prior to therapy.

The use of cannabinoids (approved agents such as dronabinol and nabilone) remains a potential therapy for the prevention of nausea and vomiting; the evidence for the use of medical marijuana remains insufficient based on the review of the panel.

The guideline thoroughly discusses the appropriate treatment of patients receiving radiation therapy and other patient scenarios. I urge you to read the entire guideline to familiarize yourself with all the recommendations and updates to the management of CINV.

## IMPLICATIONS OF COST

ASCO notes that patients with cancer are increasingly paying larger proportions of their treatment costs. Higher costs can translate into inadequate treatment of CINV, as patients may forgo paying for ordered antiemetics. Although generic formulations exist for some of the agents in our antiemetic armamentarium, many of the drugs are brand names and thus may be priced higher accordingly. If appropriate, ASCO recommends clinicians discuss cost as a critical part of shared decision-making and consider lower-priced combination therapies if they are available (Hesketh et al., 2017).

Inadequate treatment of CINV is not only a feared side effect for our patients receiving chemotherapy, but one that may lead to patients giving up potentially life-saving treatments because of CINV. And although the agents themselves can be very costly, it is significantly costly to undergo a hospitalization for the control of CINV. A recently reported abstract at the 2017 Palliative and Supportive Care in Oncology Symposium noted that in a study of 37,730 hospital discharges for nausea and vomiting, the adjusted paid amounts averaged > $15,000 (Roeland et al., 2017). The study included CINV patients as well as any cancer patients with a diagnosis of nausea and vomiting. The authors of the study note that this significant economic impact points to the need for optimal control and improved compliance with national antiemetic guidelines by oncology professionals. Most importantly, it points to the need to improve our patients’ quality of life by better control of CINV.

## OUR STAKE IN BETTER ANTIEMETIC CONTROL

As advanced practitioners, we excel in the management of symptoms and often develop and run our own clinics to take care of oncology patients with side effects from chemotherapy. We are at the front line in directing antiemetic regimens for our patients, utilizing patient risk factors, available agents, and the all-important national evidence-based guidelines for optimal care.

Poorly managed CINV is expensive, but its most obvious and troubling outcome is the damage done to our patients in terms of quality of life and compliance with future therapy. My experience with this is personal. As my brother experienced unremitting CINV with cisplatin-based therapy for his testicular cancer, he finally gave up and opted to stop therapy. I don’t want another patient to have to suffer like that. As advanced practitioners, we have the tools to do a better job. With the use of national guidelines and an ever-expanding armamentarium of antiemetic agents, let’s try to eliminate CINV once and for all.
